# Genetic and morphological evidence for a new species of the Maculatus Group of *Anopheles* subgenus *Cellia* (Diptera: Culicidae) in Java, Indonesia

**DOI:** 10.1186/s13071-019-3358-2

**Published:** 2019-03-14

**Authors:** Rusdiyah Sudirman Made Ali, Isra Wahid, Atiporn Saeung, Anchalee Wannasan, Ralph E. Harbach, Pradya Somboon

**Affiliations:** 1 Department of Parasitology, Faculty of Medicine, Hasanuddin University, Makassar, Indonesia; 2 Center of Insect Vector Study, Department of Parasitology, Faculty of Medicine, Chiang Mai University, Chiang Mai, Thailand; 3 Department of Life Sciences, Natural History Museum, Cromwell Road, London, SW7 5BD UK

**Keywords:** Java, Indonesia, ITS2, *cox*2, *Anopheles maculatus*, Genetics, Taxonomy

## Abstract

**Background:**

*Anopheles maculatus*, a species of the Maculatus Group of subgenus *Cellia* (Diptera: Culicidae), is an important vector of human malarial protozoa in Java, Indonesia. However, the identity of this species in Indonesia has been questionable because published reports and records are based mainly on morphological identification, which is unreliable for distinguishing members of the Maculatus Group due to overlapping characters.

**Methods:**

We performed morphological assessments, metaphase karyotype preparations, phylogenetic analyses of ITS2 and *cox*2 sequence data and cross-mating experiments to determine whether the Javanese form and *An. maculatus* (*s.s.*) from Thailand were conspecific.

**Results:**

The adults of the Java strain are similar to those of *An. maculatus* (*s.s.*), but the larvae and pupae exhibit significant differences. The metaphase karyotype of Javanese specimens includes a long acrocentric X chromosome and a small telocentric Y chromosome, which are distinct from other members of the Maculatus Group. Cross-mating of the Java strain with *An. maculatus* (*s.s.*) revealed genetic incompatibility. Phylogenetic analysis of ITS2 and *cox*2 sequences revealed that the Java strain forms a single clade that is distinct from clades of other members of the group (Kimura 2-parameter, K2P, genetic distances 3.1–19.2% and 1.6–9.6%, respectively).

**Conclusions:**

This study provides evidence that the Javanese form of *An. maculatus* is not conspecific with *An. maculatus* (*s.s.*) and constitutes a previously unrecognized species of the Maculatus Group.

## Background

The Maculatus Group [1] of the Neocellia Series of subgenus *Cellia* Theobald of *Anopheles* Meigen includes nine formally recognized species: *An. dispar* Rattanarithikul & Harbach, *An. dravidicus* Christophers, *An. greeni* Rattanarithikul & Harbach, *An. maculatus* Theobald, *An. notanandai* Rattanarithikul & Green, *An. pseudowillmori* (Theobald), *An. rampae* Harbach & Somboon, *An. sawadwongporni* Rattanarithikul & Green and *An. willmori* (James) [2]. Species of this group have overlapping morphological characters, and therefore multiple methods of investigation have been used to distinguish and define them, including cytogenetics [3–5], comparative morphology [1, 6], crossing mating experiments [7, 8] and phylogenetic analysis [9–11].

Sinka et al. [12] provided a summary of information on the distribution and bionomics of the Maculatus Group. Members of the group are usually found in or near hilly forested terrain, as well as high mountainous areas. The immature stages are found in a variety of habitats, such as ponds, lakes, stream margins, stream pools, ground pools, sand pools, rock pools, marshes, river beds, rice fields, etc. The species have overlapping and disparate distributions and are variously involved in the transmission of human malarial parasites in tropical and subtropical areas of Asian countries. Among members of the group, the nominotypical species (*An. maculatus*) appears to be the most widely distributed species, ranging from Afghanistan, Pakistan and India eastward to the western Pacific islands, including Taiwan, the Indonesian archipelago and Timor Leste. It is absent from the Philippines where *An. dispar* and *An. greeni* are present [6, 13, 14].

Human malarial parasites have been detected in *An. maculatus* across its distribution, but its role in transmission to humans is well documented only in peninsular Malaysia [14, 15]. *Anopheles willmori* is a primary vector of human malarial protozoa in the highlands of Nepal [16]. *Anopheles pseudowillmori* is a vector in southern Tibet [17], a secondary vector in western Thailand [18] and a suspected vector in Bhutan [19]. *Anopheles sawadwongporni* may be a secondary vector in Thailand and Vietnam [20–22]. *Anopheles dispar* and *An. greeni* are regarded as secondary vectors in the Philippines [13]. It is not known whether *An. dravidicus*, *An. notanandai* and *An. rampae* may play a role in malaria transmission.

Malaria is a significant public health problem throughout the Indonesian archipelago. Over 200,000 malaria cases were reported in Indonesia in 2016 [23]. Of about 80 *Anopheles* species recorded in Indonesia, 21 have been incriminated as primary and secondary vectors of malaria, but their roles in malaria transmission vary across the archipelago [24–27]. In Java, *An. maculatus* is recorded as one of the important vectors, but it is of little or no medical importance on other islands of Indonesia due to its more zoophilic behavior and normally low human-biting densities [25, 28]. Other malaria vector species in Java include *An. aconitus* Dönitz, *An. balabacensis* Baisas, *An. flavirostris* (Ludlow), members of the *An. sundaicus* and *An. subpictus* complexes, and *An. vagus* Dönitz [25].

Published reports and records of *An. maculatus* in Java and other islands of Indonesia are based mainly on morphological identifications; hence, it is not possible to know with certainty if it is conspecific with continental populations of the species [9–11]. For many years, it was pointed out that the taxonomic status of *An. maculatus* in Indonesia is questionable and may represent an undescribed species in the Maculatus Group [6, 25, 29].

In this study, we performed morphological assessments, metaphase karyotype preparations, cross-mating experiments and phylogenetic analyses of DNA sequence data to provide, for the first time, clear evidence that *An. maculatus* in Java is a distinct species of the Maculatus Group.

## Methods

### Mosquitoes and morphological identification

Specimens of a stenogamous laboratory strain of *An. maculatus* from Java (Java strain) [29] maintained at the Vector and Reservoirs Research Institute in Salatiga, Central Java, were used to establish a colony in the Department of Parasitology, Hasanuddin University, Indonesia. This strain was used for examination of metaphase karyotypes and cross-mating with specimens from a laboratory colony of *An. maculatus* (*s.s.*) (Thailand strain), which was established with specimens collected in Mae Sarieng District in Mae Hong Son Province of northwestern Thailand and maintained at the Office of Disease Prevention and Control, Region 1, Chiang Mai two years prior to the present study. The specimens of the Thailand strain exhibit the characters that define all life stages of *An. maculatus* (*s.s.*) [1, 6, 11], and their ITS2 sequences fall in the clade with sequences previously generated for this species (see the “Results” below). Specimens of both strains were transferred to the insectary of the Department of Parasitology, Faculty of Medicine, Chiang Mai University, Thailand for rearing and study. The insectary was maintained at about 27 °C and 70% relative humidity with a photoperiod of 12:12 h L:D. Females of the Thailand and Java strains and feral females collected in a cow-shed in Kulonprogo, Central Java, were used for DNA sequencing.

Morphological features of adult mosquitoes were examined under a stereomicroscope. Mature larvae were killed with hot water (60–65 °C) and preserved in 80% ethanol. Larval and pupal exuviae were collected within 24 h after molting or emergence and preserved in 80% ethanol. They were mounted on microscope slides with Hoyer’s medium (Neo-shigaral, Shiga Konchu Fukyusha, Tokyo, Japan) or Euparal (Waldeck, Germany). Eggs and larval and pupal setae were examined under a bright-field compound microscope (Olympus CX31) using 10× and 40× objective lenses. Available morphological keys [1, 6, 30] were used to confirm identification of the species. Photographs were taken with a digital camera (Olympus E-330). The morphological terminology and abbreviations follow the Anatomical Glossary of the online Mosquito Taxonomic Inventory [31].

### Metaphase karyotypes

Metaphase chromosomes were prepared from the brain ganglia of 10 fourth-instar larvae of the Java strain, using techniques described by Saeung et al. [32]. Identification of karyotypic forms followed the method of Baimai et al. [4].

### Cross-mating experiments

Reciprocal crosses between the Java and Thailand strains were carried out to determine genetic compatibility. Pupae were sexed by observing the shape and size of the genital lobe ([31], http://mosquito-taxonomic-inventory.info/simpletaxonomy/term/6691) and kept separately until emergence of adults. Virgin blood-fed females were mated with males using the artificial mating technique [33]. Following mating, each female was isolated in an oviposition cup and provided with a cotton plug wetted with a 10% sucrose solution. Eggs were counted and allowed to hatch. Following oviposition, females were dissected to check for spermatozoa in their spermathecal capsules, and eggs from un-inseminated females were discarded. Newly hatched larvae from each egg batch were counted and placed in rearing trays. They were reared in dechlorinated water and fed with a finely ground fish food until pupation. Pupae were removed daily, sexed and placed separately in cups until emergence of adults. The emergent rates of F_1_ hybrid adults were noted. The testes and ovaries of hybrids were dissected to check fertility. The fertility and viability of hybrids was determined by backcrosses with the Java strain.

Egg batches with no or little hatching were allowed to stand for another 3 days, and afterwards examined for the development of embryos. To check embryonation, both hatched and unhatched eggs were transferred onto a drop of water on a microscope slide, covered with a coverslip, gently pressed with the blunt end of a pen to break the egg chorion, and examined under a microscope for embryo formation.

### DNA extraction, amplification and sequencing

Genomic DNA was extracted from whole mosquitoes or legs of individual adult mosquitoes using Pure Link™ Genomic DNA Mini Kit (Invitrogen by Thermo Fisher Scientific, USA) according to manufacturer’s instructions. The specimens, without legs, were retained for morphological examination. A product of approximately 450 bp of the ITS2 region of rDNA was amplified by PCR using the primers 5.8F (5′-TGT GAA CTG CAG GAC ACA TG-3′) and 28R (5′-ATG CTT AAA TTT AGG GGG TA-3′) [11]. Each PCR reaction was carried out in a 20 µl volume containing 1 µl of DNA, 0.8 µM of each primer, 1.2 mM MgCl_2_, 1.6 mM dNTP mix and 0.08 U of Platinum^®^Taq DNA polymerase in 1× PCR buffer. Thermals cycling conditions for ITS2 included an initial denaturation at 95 °C for 2 min, 35 cycles at 95 °C for 1 min, 55 °C for 1 min, and 72 °C for 2 min and a final extension step at 72 °C for 5 min.

The mitochondrial *cox*2 gene was amplified using the primers SCTL2-J-3037 (5′-ATG GCA GAT TAG TGC AAT GA-3′) and TK-N-3785 (5′-GTT TAA GAG ACC AGT ACT TG-3′) [34]. PCR reactions were carried out in a 20 μl volume containing 0.4 U of Platinum^®^Taq DNA polymerase, 1× of PCR buffer, 1.5–3.0 mM of MgCl_2_, 0.2 mM of each dNTP, 0.2 μM of each primer and 1 μl of extracted DNA. The amplification profile comprised initial denaturation at 95 °C for 5 min, 35 cycles at 95 °C for 30 s, 45 °C for 30 s and 72 °C for 30 s, and a final extension step at 72 °C for 5 min. The amplified products were electrophoresed in 2% agarose gels and stained with ethidium bromide. PCR products were purified using the illustra™ ExoProStar™ 1-Step (GE Healthcare Life Sciences, UK) and sequenced using a 23 ABI 3730XLs sequencer (Macrogen, South Korea).

### Phylogenetic analysis

The ITS2 and *cox*2 sequences obtained during this study were compared with those of *An. maculatus* (*s.s.*) and other members in the Maculatus Group on GenBank using the Basic Local Alignment Search Tool (BLAST, http://blast.ncbi.nlm.nih.gov/Blast.cgi). *Anopheles stephensi* Liston was used as the outgroup taxon. Sequences of ITS2 were manually aligned using the CLUSTALW [35] and edited manually in MEGA version 7.0. Gap sites were excluded from the analysis. Construction of Neighbor-joining trees and the bootstrap test with 1000 replications were conducted with MEGA version 7.0 [36]. Genetic distances were estimated by using the Kimura two-parameter (K2P) method [37].

## Results

### Morphology

**Fig. 1:**
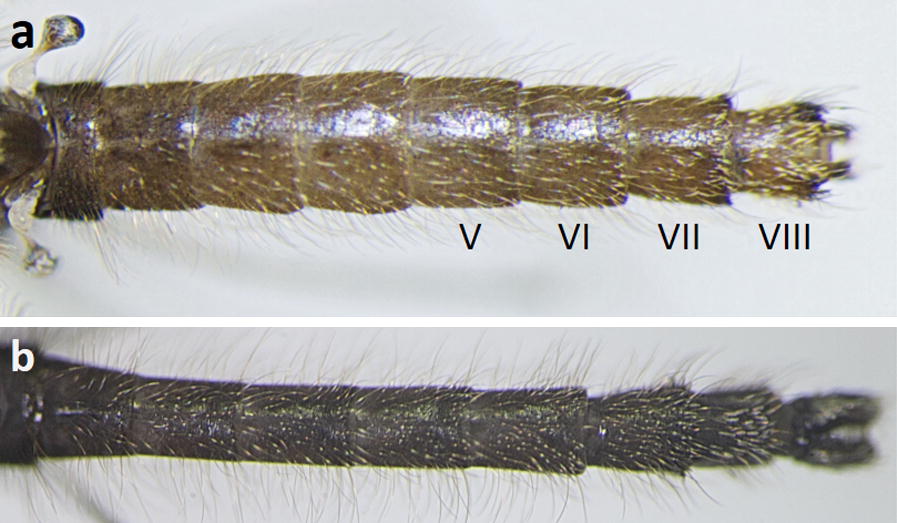
Abdominal terga of a female (**a**) and male (**b**) of the *An. maculatus* Java strain

**Fig. 2:**
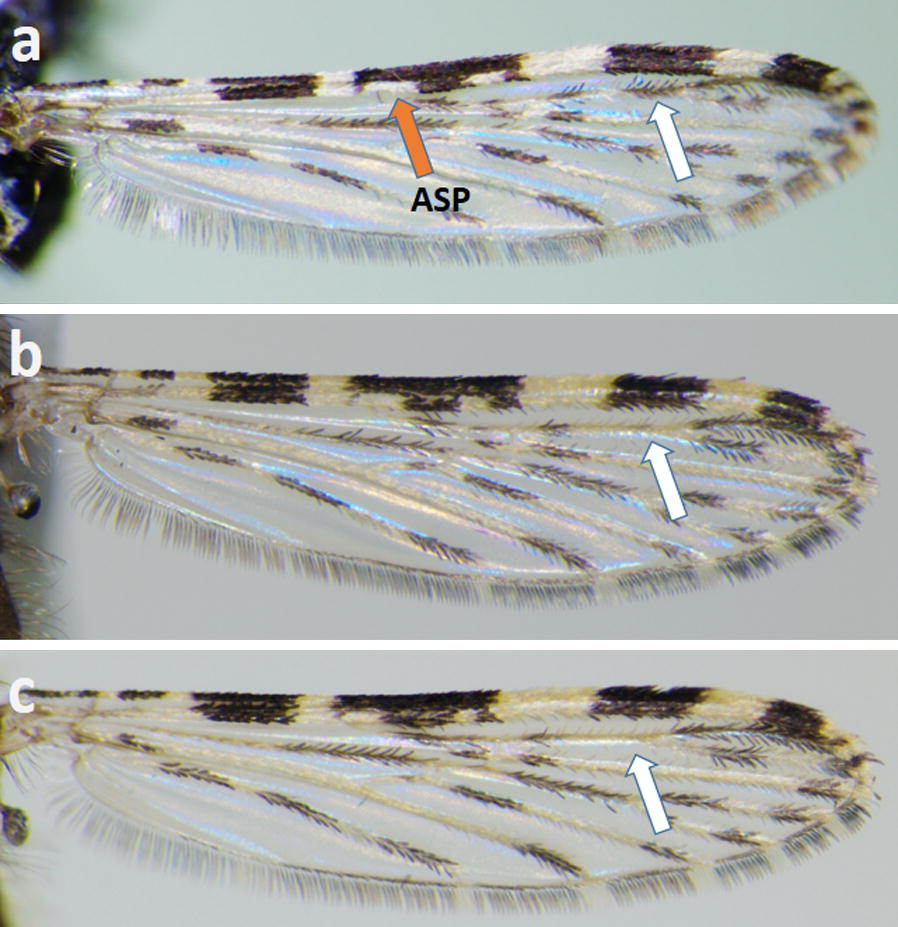
Variation of wing scaling in specimens of the *An. maculatus* Java strain. The presence/absence of a distal dark spot on vein R_2+3_ is indicated by a white arrow (**a-c**). The accessory sector pale spot (ASP) is confined to vein R_1_ indicated by a red arrow (**a**)

Fifty-two females and 27 males of the Java strain, and six wild caught females from Java were examined. In general, females and males of the strain look similar to *An. maculatus* (*s.s.*), in particular abdominal terga II–IV are without scales; terga V and VI are without scales or have sparse pale falcate scales on the posterior margins; terga VII and VIII are largely or posteriorly covered with narrow pale spatulate scales; the posterolateral corners of terga VI–VIII, and rarely tergum V, bear patches of dark scales (Fig. 1a, b). Variation of wing scaling has been found in both the laboratory Java strain and feral females, in particular, vein R_2+3_ has one dark spot or two dark spots on at least one wing (Fig. 2a-c; Table 1). The two spots occasionally join to form a single large spot, or are reduced to a few dark scales near the furcation. In both feral and laboratory females, the accessory sector pale spot is confined to vein R_1_ and is never present on the subcosta and costa. Palpomere 3 is completely black-scaled.

**Table 1:** Frequency of wing-scale characters of the laboratory Java strain and wild-caught females from Java

Characters	Wild females (*n* = 6)	Laboratory strain	
		Females (*n* = 52)	Males (*n* = 27)
Vein R_2+3_ with 2 dark spots on both wings (%)	33.3 (2)	23.1 (12)	48.1 (13)
Vein R_2+3_ with 2 dark spots on one wing (%)	0 (0)	28.8 (15)	22.2 (6)
Vein R_2+3_ with pale spots on both wings (%)	66.7 (4)	48.1 (25)	29.7 (8)
Presector dark spot (PSD) on vein R as long as PSD on subcosta and costa on both wings (%)	33.3 (2)	69.2 (36)	92.6 (25)
Presector dark spot (PSD) on vein R as long as PSD on subcosta and costa on one wing (%)	50.0 (3)	7.7 (4)	3.7 (1)
Presector dark spot (PSD) on vein R shorter than PSD on subcosta and costa on both wings (%)	16.7 (1)	23.1 (12)	3.7 (1)

**Fig. 3:**
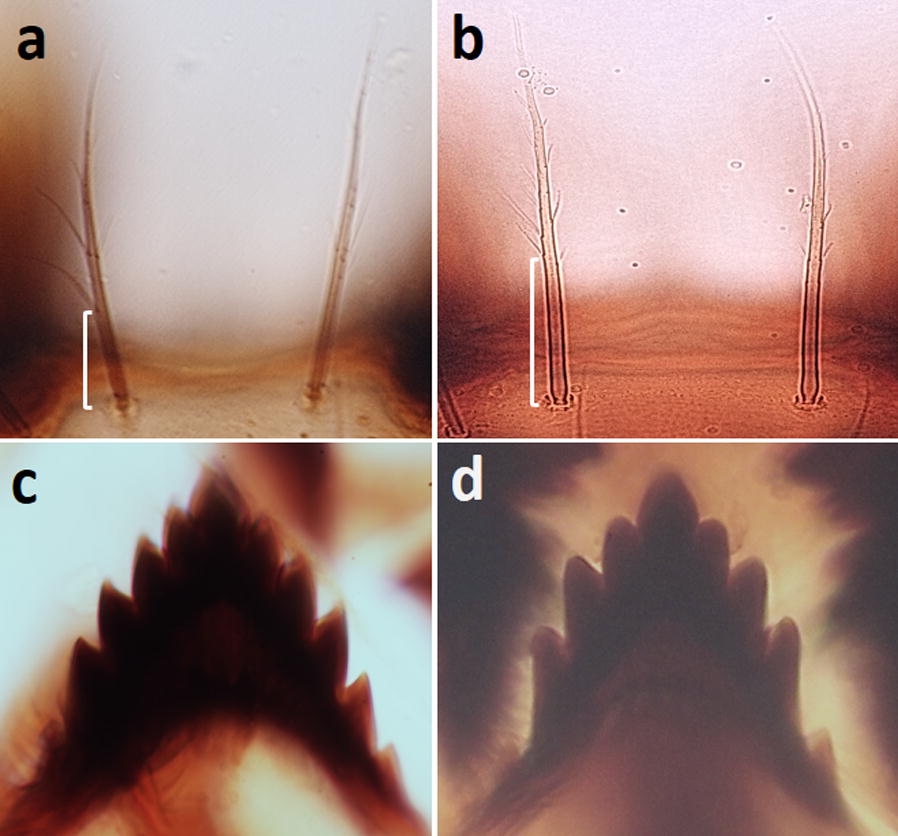
Comparison of seta 2-C and dorsomentum of the head of larvae. **a** Seta 2-C of the Java strain with long, strong lateral barbs (aciculae) arising near the base. **b** Seta 2-C of the Thailand strain with short, weak barbs arising far from the base. **c** Dorsomentum of the Java strain with 11 teeth. **d** Dorsomentum of the Thailand strain with nine teeth

**Fig. 4:**
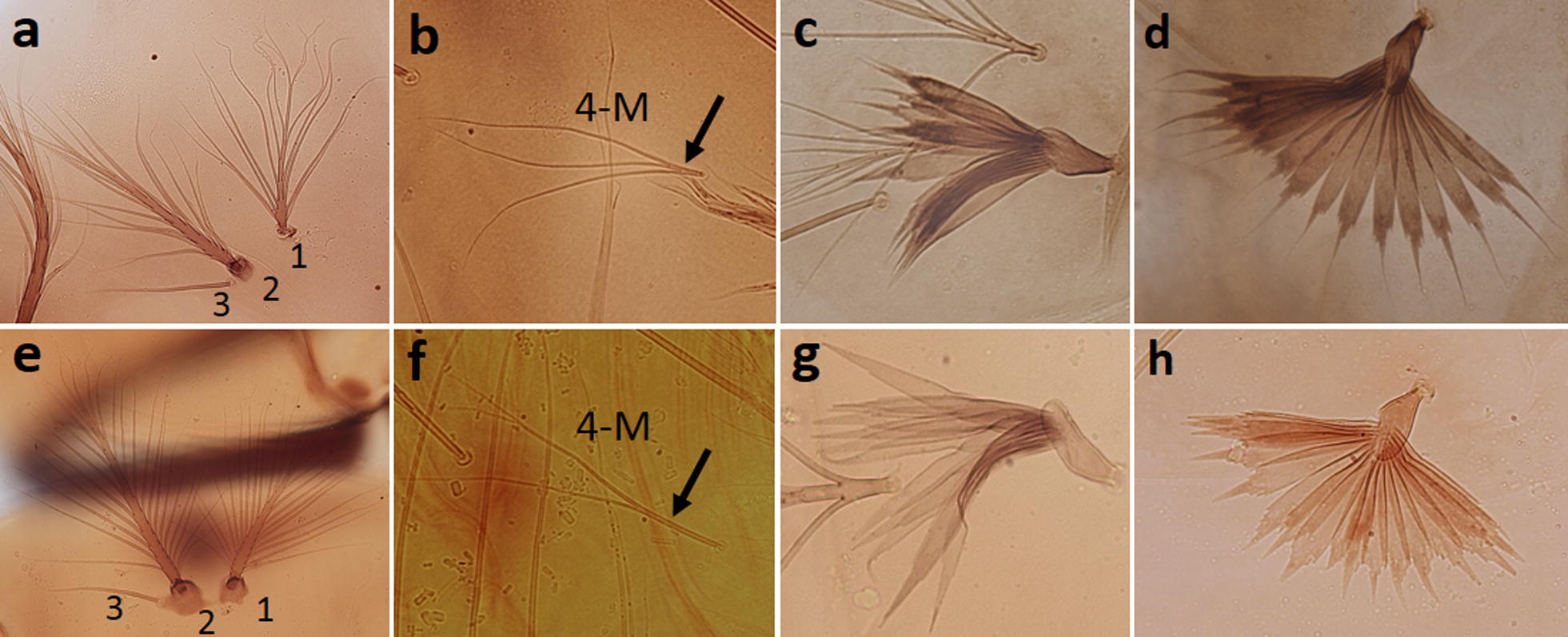
Setae of *An. maculatus* larvae from Java (**a-d**) and Thailand (**e-h**). **a**, **e** Thoracic setae 1–3-P. **b**, **f** Thoracic seta 4-M. **c**, **g** Abdominal palmate seta 1-II. **d**, **h** Abdominal palmate seta 1-IV

Ten larvae of the Java strain were examined and compared with *An. maculatus* (*s.s.*) (Thailand strain). In general, the Javanese larvae exhibit characters of the Maculatus Group, but differ from *An. maculatus* (*s.s.*) as follows: seta 2-C has long, strong lateral barbs (aciculae) arising near the base (on basal 0.21, 0.11–0.31) (Fig. 3a), but that of *An. maculatus* (*s.s.*) has short, weak lateral barbs arising far from the base (on basal 0.39, 0.34–0.43) (Fig. 3b); the dorsomentum of the head has 5 teeth on either side of the median tooth (total 11 teeth) (Fig. 3c), whereas *An. maculatus* (*s.s.*) has 4 teeth on either side of the median tooth (total 9 teeth) (Fig. 3d); seta 1-P has 11–20 branches and the stem of seta 1-P is usually weaker than the stem of seta 2-P (Fig. 4a), but in *An. maculatus* (*s.s.*) seta 1-P has > 20 branches and the stem of seta is as strong as that the stem of seta 2-P (Fig. 4e); seta 3-P is separated from or proximal to the tubercle supporting seta 2-P, but that of *An. maculatus* (*s.s.*) is borne on a small tubercle joined to the tubercle supporting 2-P; the basal stem of seta 4-M is 3–5 times as long as its width (Fig. 4b), whereas it is > 5 times as long in *An. maculatus* (*s.s.*) (Fig. 4f); most leaflets of abdominal palmate seta 1-II have distinctly serrated shoulders and a long filament (Fig. 4c), whereas those of *An. maculatus* (*s.s.*) rarely have distinctly serrated shoulders (Fig. 4g); leaflets of abdominal seta 1-III–VI have long slender, sharply pointed filaments that are one-third to one-half the length of the blade (Fig. 4d), whereas those of *An. maculatus* have short filaments that are one-fourth the length of the blade (Fig. 4h).

**Fig. 5:**
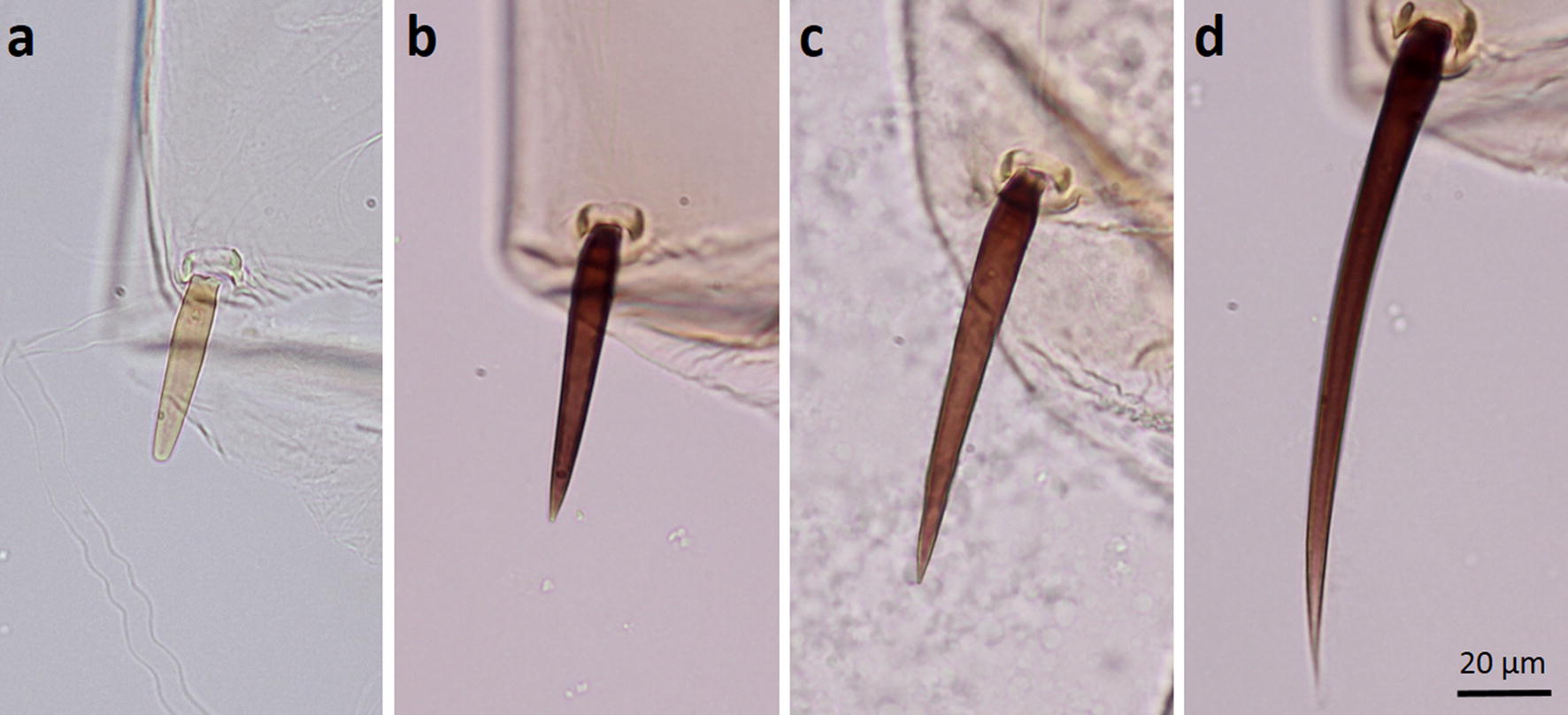
**a** Abdominal seta 9-IV of the pupa of *An. maculatus* (*s.s*.) Thailand strain. **b**, **c** Abdominal seta 9-IV of the pupa of the *An. maculatus* Java strain. **d** Abdominal seta 9-V of the pupa of the *An. maculatus* Java strain

Ten pupal exuviae of the Java strain were examined. In general, they are similar to those of *An. maculatus* (*s.s.*) and no clear differences in setal branching were observed. Both strains have seta 9-II very short, blunt and transparent; seta 9-III is slightly longer than seta 9-II, blunt and darkly pigmented. However, a significant difference was observed in the development of abdominal seta 9-IV, which is short and blunt in *An. maculatus* (*s.s.*) (Fig. 5a) and horn-like, pointed or long and sharply pointed in the Javanese pupae (Fig. 5b, c). The ratio of lengths of seta 9-IV/9-V (Fig. 5b-e) of the Javanese pupae is 0.38–0.68 (mean 0.48), whereas in *An. maculatus* (*s.s.*) it is 0.22–0.38 (mean 0.28).

The eggs of the Java strain are generally similar to those of other members in the Maculatus Group, except *An. sawadwongporni*, in having the frill incomplete in the middle on both sides (figure not shown).

### Metaphase karyotype

**Fig. 6:**
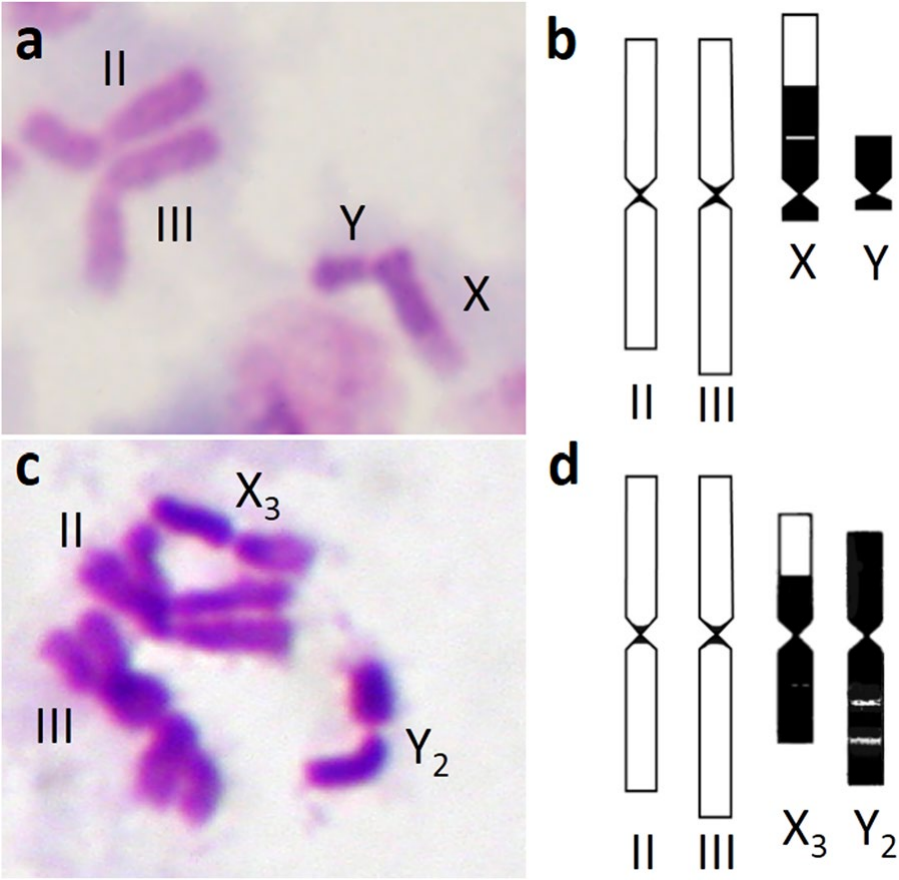
Metaphase karyotypes from larval neuroblast cells. **a**, **b** *An. maculatus* Java strain: **a** Giemsa stain showing a long acrocentric X chromosome, a small telocentric Y chromosome and autosomes II and III and **b** diagrammatic representation. **c**, **d** *An. maculatus* Thailand strain: **c** large submetacentric X_3_ and Y_2_ chromosomes and autosomes II and III and **d** diagrammatic representation. Only one set of autosomes II and III is present. Variable heterochromatic portions are indicated in black. The centromeres are indicated by constrictions of each chromosome. Chromosome lengths, arm ratios and heterochromatic parts are shown in proportion

The metaphase karyotype was observed in the F_6_ progeny of the Java strain compared with the Thailand strain. The Java strain appears to comprise one genotypic form (Fig. 6a, b). The X chromosome is acrocentric, slightly longer that the long arm of autosome III and consists of a large block of centromeric heterochromatin and an euchromatic portion that is slightly shorter than the heterochromatic block. The heterochromatic Y chromosome is obviously small, telocentric and slightly shorter than the length of the heterochromatic block of the X chromosome. The autosomes possess a very limited amount of pericentric heterochromatin. By contrast, the X and Y chromosomes of the Thailand strain are large submetacentric (Fig. 6c, d), and similar to X_3_ and Y_2_ chromosomes of *An. maculatus* (form B) as described by Baimai et al. [4].

### Cross-mating

**Fig. 7:**
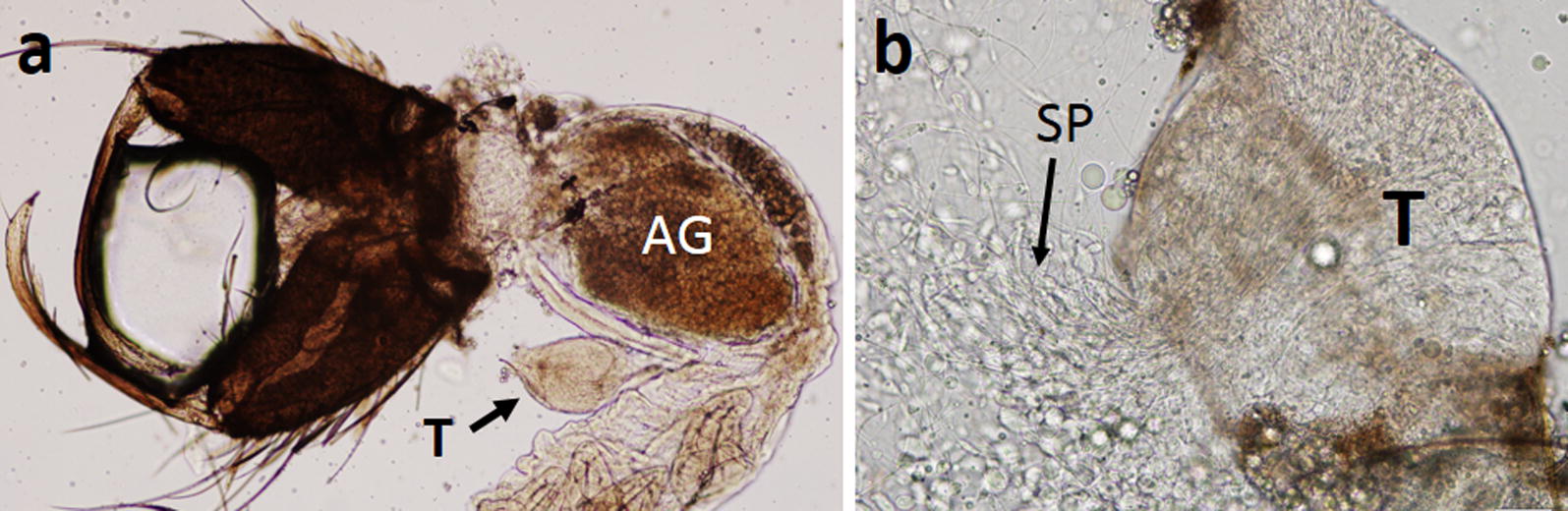
Reproductive organs of F_1_ hybrids derived from crosses between *An. maculatus* Java strain and *An. maculatus* Thailand strain. **a** Accessory glands (AG) and sterile testis (T). **b** Testes of hybrid male derived from backcross showing abnormal spermatozoa (SP) with enlarged heads

Cross-mating between the Java and Thailand strains revealed genetic incompatibility. The resulting fertility and viability are summarized in Table 2. Reciprocal crosses in both directions yielded F_1_ hybrids, but egg hatchability and adult emergence were low. The control cross (Java × Java) resulted in normal fertility and viability. All hybrid females examined appeared to have normal ovaries. Dissection revealed that all hybrid males were completely sterile, but the accessory glands looked normal (Fig. 7a). Therefore, these hybrid males were not used for backcrossing. Backcrosses between F_1_ hybrid females and males of the Java strain resulted in low egg hatchability and adult emergence. All males that emerged were partially sterile, having abnormal spermatozoa with enlarged heads (Fig. 7b) and inactive spermatozoa, while the ovaries of females appeared to be normal.

**Table 2:** Crossing and backcrossing combinations of *An. maculatus* Java strain (JV) and *An. maculatus* (*s.s*.) Thailand strain (TH)

Crosses		No. of broods	Mean no. of eggs per oviposition (total)	Percent embryonation^a,b^	Percent eggs hatched^a^	Percent emergence^a^ (no.)		Percent female and male with abnormal ovaries and testes, respectively (no. dissected)	
Female	Male					Female	Male	Female	Male
JV	JV	1	100	100	100	34.0 (34)	47.0 (47)	0 (5)	0 (5)
JV	TH	5	55.2 (276)	66.3	52.2	15.9 (44)	16.3 (45)	0 (7)	100 (3)^c^
TH	JV	1	63	19.1	17.5	6.35 (4)	6.35 (4)	nd	nd
Backcross									
(JV × TH)F_1_	JV	5	55.6 (278)	53.6	35.6	14.4 (40)	14.7 (41)	0 (10)	100 (12)^d^

^a^Calculated from the total number of eggs

^b^Calculated from the number of larvae that emerged plus the number of dead embryos inside unhatched eggs

^c^All males dissected were completely sterile

^d^Most spermatozoa were abnormal (with enlarged heads) or inactive

*Abbreviation*: nd, not determined

### DNA sequences and phylogenetic analysis

The ITS2 region was sequenced from five females of the Java strain, two without a distal dark spot on vein R_2+3_ and three with such a spot. Three wild-caught females from Java were also sequenced, but unfortunately without noting the wing scales. The *cox*2 region was sequenced for the three feral females and four of the five females of the Java strain. The ITS2 region was sequenced from two females of the Thailand strain. The sequences are deposited in the DDBJ/EMBL/GenBank nucleotide sequence database under the accession numbers MK204640-MK204649 (ITS2) and MK236365-MK236371 (*cox*2).

**Fig. 8:**
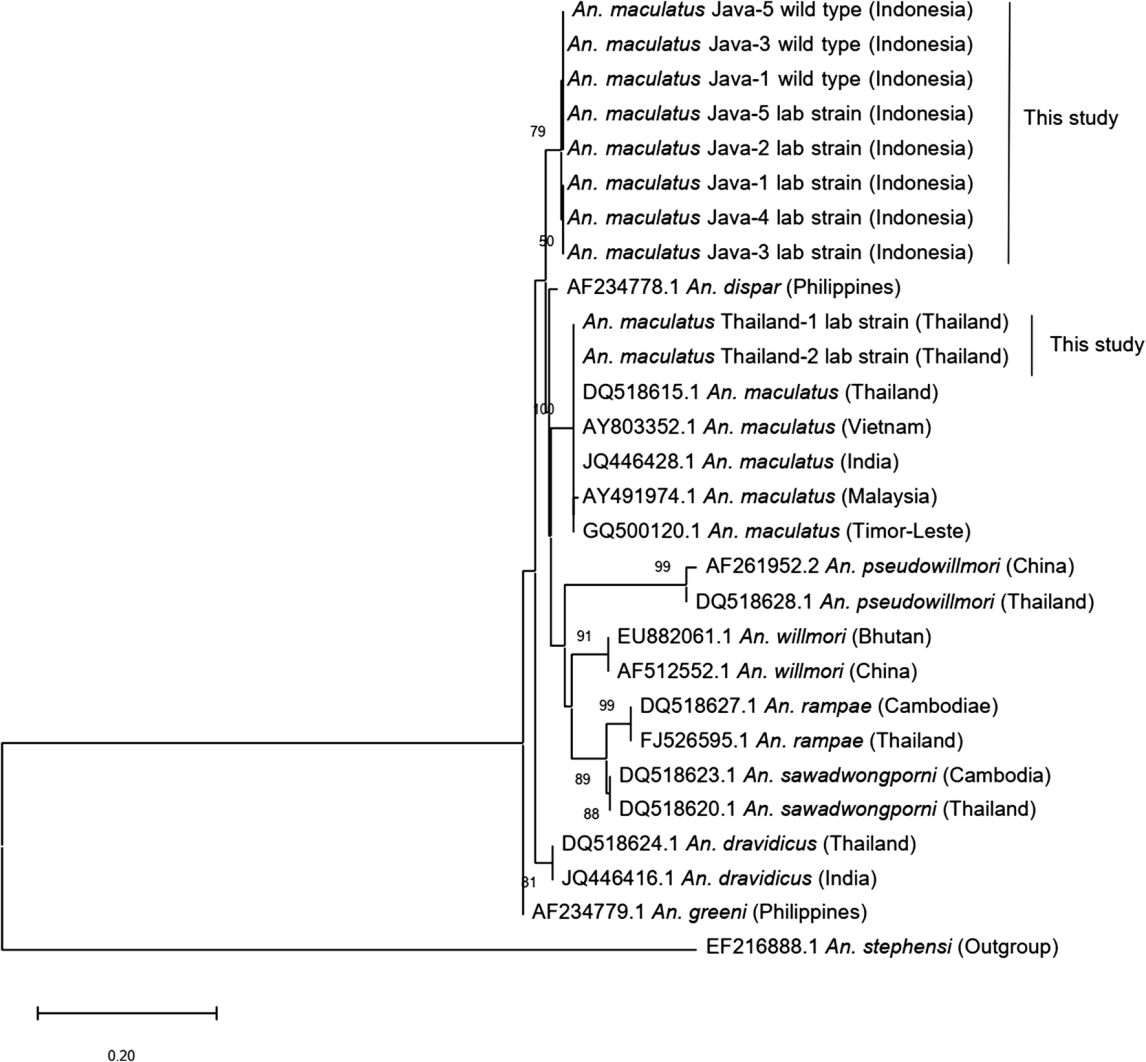
Phylogenetic tree derived from ITS2 sequences of specimens from the laboratory colony of the Java strain, wild-caught females from Java, *An. maculatus* Thailand strain and GenBank sequences of other members of the Maculatus Group, with *An. stephensi* as the outgroup

**Fig. 9:**
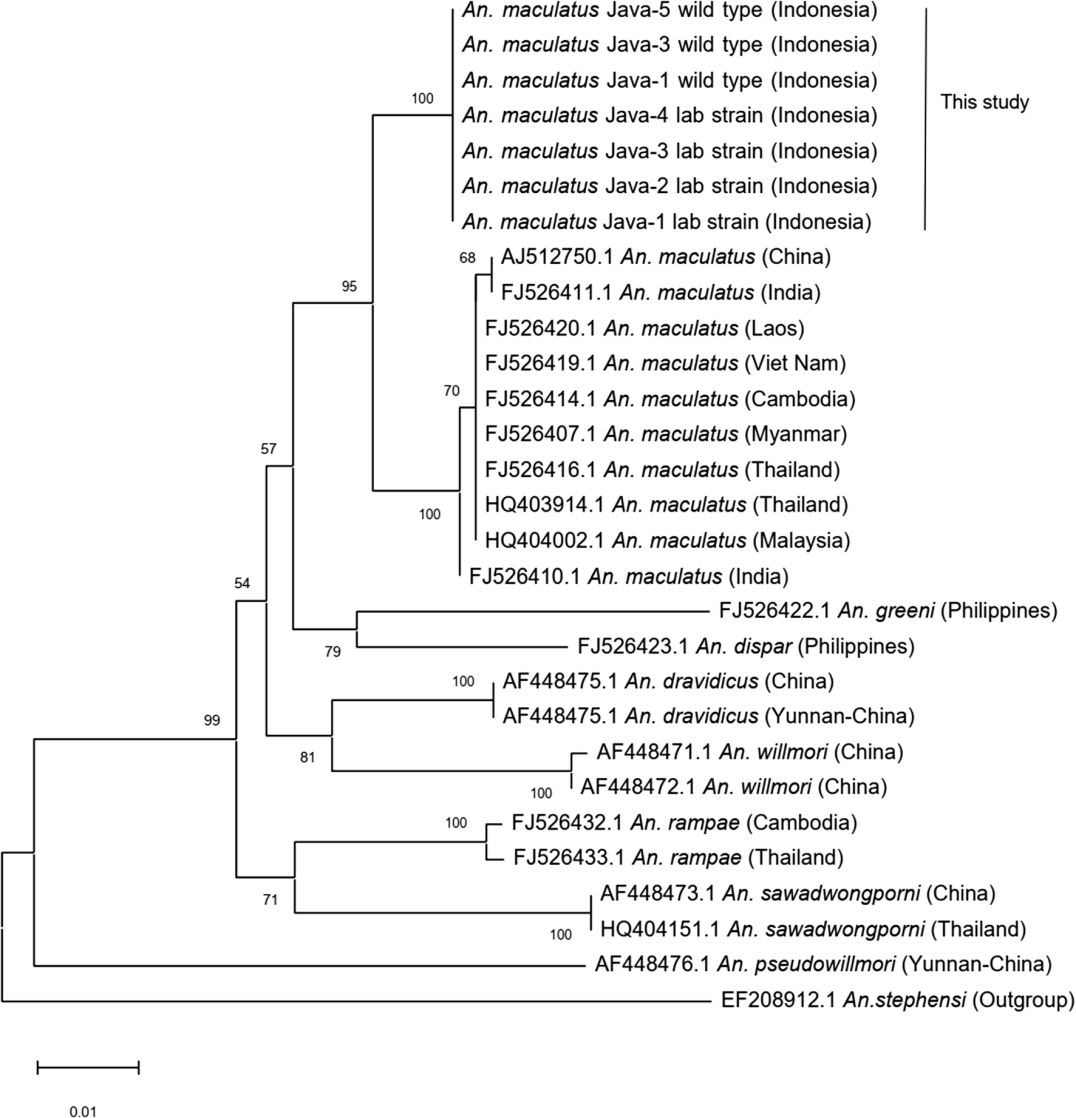
Phylogenetic tree derived from *cox*2 sequences of specimens from the laboratory colony of the Java strain, wild-caught females from Java and GenBank sequences of other members of the Maculatus Group, with *An. stephensi* as the outgroup

The ITS2 sequences (334 bp) of the Java strain, with and without a distal dark spot on vein R_2+3_, and those of the wild-caught females were identical or very similar (K2P genetic distance 0–0.3%), whereas all of the *cox*2 sequences (620 bp) were identical. The ITS2 sequences of two specimens of the Thailand strain were also identical to one another. Phylogenetic analysis of the ITS2 and *cox*2 sequences (Figs. 8, 9) revealed that specimens of the Java strain form a single clade that is distinct from *An. maculatus* (*s.s.*) (Thailand strain) and other species of the Maculatus Group (K2P, ITS2: 3.1–19.2%; *cox*2: 1.6–9.6%). Intraspecific variation of the ITS2 and *cox*2 sequences of *An. maculatus* (*s.s.*) from continental Asian countries, as indicated in Figs. 8 and 9, was very low (K2P, ITS2: 0–0.6%; *cox*2: 0–0.3%). Comparison of the ITS2 sequences reveals that the Java clade seems to be most closely related to *An. dispar* (K2P 3.1–3.4%), followed by *An. maculatus* (*s.s.*) (K2P 4.6–5.6%) and *An. greeni* (K2P 5.0–5.3%). However, comparison of the *cox*2 sequences indicates that the Java clade is most closely related to *An. maculatus* (*s.s.*) (K2P 1.6–1.8%), followed by *An. dispar* (K2P 4.0%) and *An. dravidicus* (K2P 4.3%).

## Discussion

The results of the present study provide unambiguous evidence that *An. maculatus* in central Java is not conspecific with *An. maculatus* (*s.s.*) and is a distinct species of the Maculatus Group. Morphologically, the adult and larval stages have morphological characters that overlap with those of various species of this group. The adults have pale scaling and patches of dark scaling on the posterolateral corners of abdominal terga VI–VIII that is similar to the scaling of *An. dispar*, *An. greeni* and *An. maculatus* (*s.s.*) [6]. The wings of Javanese adults either have or lack a distal dark spot near the furcation of vein R_2+3_. Specimens without the spot are difficult to separate from *An. dispar*, *An. greeni* and *An. maculatus* (*s.s.*). However, about half of specimens of *An. dispar* and *An. greeni* have an accessory sector pale spot on the costa and subcosta, which is absent in Javanese specimens and other members of the Maculatus Group. The presence of a distal dark spot on vein R_2+3_ is common in species of the Sawadwongporni Subgroup, i.e. *An. notanandai*, *An. rampae* and *An. sawadwongporni*, but those species differ in having abdominal terga II–VIII densely covered with pale spatulate scales and posterolateral corners of terga II and IV usually with a few black scales [1, 38]. We consider that the two wing forms found in Javanese specimens are conspecific because both forms have similar or identical ITS2 and *cox*2 sequences. The absence of the distal dark spot on vein R_2+3_ was found in about 15% of wild-caught adults of *An. rampae* [38] and noted in *An. sawadwongporni* [1]. Therefore, variation of the wing scaling in Javanese specimens is considered to be due to intraspecific variation that can confound morphological identification. However, as only six wild-caught females were available for study, additional specimens are needed to assess the variable presence of the distal dark spot in Javanese populations.

Larvae of the Java strain differ mainly from other members of the Maculatus Group in having longer and stronger lateral barbs (aciculae) near the base of seta 2-C (Fig. 3a), and in having the dorsomentum with 11 teeth (Fig. 3c). As stated by Christophers [39], seta 2-C of *An. maculatus* (*s.s.*) is very finely frayed on the distal two-thirds, and the dorsomentum has four teeth on either side of median tooth (total of 9 teeth) [6]. Compared with *An. maculatus* (*s.s.*) [6], the stem of seta 1-P of Javanese larvae is usually weaker with fewer branches, and seta 3-P is not borne on a tubercle joined to the tubercle supporting seta 2-P. Rattanarithikul et al. [30] used seta 4-M to characterize species of the Sawadwongporni Subgroup: the basal stem of this seta is short and no longer than four times its width, but it is five or more times longer in other members of the Maculatus Group. The basal stem of this seta in the Javanese larvae is usually four to five, occasionally six, times its width, which may confuse identification. However, in species of the Sawadwongporni Subgroup, as well as *An. maculatus* (*s.s.*) and *An. dravidicus* of the Maculatus Subgroup, the filaments of the leaflets of palmate seta 1-III–VI are distinctly short. In contrast, the filaments are long in the Javanese larvae, as well as larvae of *An. dispar*, *An. greeni*, *An. pseudowillmori* and *An. willmori*, which can confuse identification, particularly if Javanese specimens have seta 4-M with a slightly long basal stem. The leaflets of palmate seta 1-II have distinct shoulders in larvae of the Java strain, *An. dispar*, *An. greeni* and *An. pseudowillmori*, but the shoulders are not as distinctly developed in the other members of the Maculatus Group [6, 30]. Therefore, identification of the Javanese form is possible using the combination of larval characters. Primary differences in the adults and larvae of the Java strain and the Sawadwongporni Subgroup are summarized in Table 3.

**Table 3:** Summary of primary morphological differences between adults and larvae of Javanese *Anopheles maculatus* and members of the Sawadwongporni Subgroup

Stage	Character	Javanese *An. maculatus*	Sawadwongporni Subgroup
Adult	Abdomen	Abdominal terga VI–VIII covered with pale scales and patches of dark scales on the posterolateral corners	Abdominal terga II–VIII densely covered with pale scales and posterolateral corners of terga II and IV usually with a few black scales
	Wing	Either have or lack a distal dark spot near the furcation of vein R_2+3_	Usually have a distal dark spot near the furcation of vein R_2+3_
Larva	Head		
	Seta 2-C	With long, strong lateral barbs (aciculae) arising near the base	With short, weak lateral barbs arising far from the base
	Dorsomentum	11 teeth	9 teeth
	Thorax		
	Stem of seta 1-P	Weaker than stem of seta 2-P	As strong as stem of seta 2-P
	Seta 3-P	Separated from or proximal to the tubercle supporting seta 2-P	Borne on the tubercle supporting seta 2-P
	Seta 4-M	Basal stem short, usually 3-6 times its width	Basal stem no longer than four times its width
	Abdomen		
	Seta 1-II	Leaflets usually with long, sharply pointed filaments and distinct serrated shoulders	Leaflets with short filaments, and rarely with distinct serrated shoulders
	Seta 1-III–VI	Leaflets with long slender, sharply pointed filaments, 1/3–1/2 as long as blade	Leaflets with short slender filaments, about 1/4 as long as blade

Pupae of Javanese specimens differ from pupae of *An. maculatus* (*s.s.*) in having seta 9-IV longer and more sharply pointed. This seta is shorter and blunt in pupae of *An. maculatus* (*s.s.*). The ratio of the lengths of seta 9-IV/9-V in Javanese pupae (0.38–0.68, mean 0.48) is greater than in pupae of *An. maculatus* (*s.s.*) (0.22–0.38, mean 0.28), which is nearly to the same as a previous report (0.18–0.38, mean 0.27) [6]. However, this ratio is close to that of *An. dispar* (0.30–0.71, mean 0.52) and overlaps with that of *An. greeni* (0.56–0.83, mean 0.72) [6]. Thus, variation in the length of seta 9-IV makes it difficult to separate the pupae of the Javanese strain and these two species.

Metaphase karyotypes of members of the Maculatus Group were described by Baimai et al. [4]. Similar to the Java strain, telocentric or acrocentric sex chromosomes are only found in *An. dravidicus*, *An. pseudowillmori* and *An. sawadwongporni*, whereas those of the other members of the group are large submetacentric chromosomes. The acrocentric X chromosome of the Java strain appears to be longer than the X chromosome of those three species. The small telocentric Y chromosome is shorter than the Y chromosome of *An. dravidicus* and *An. sawadwongporni*, but is similar to that of *An. pseudowillmori*. The two autosomes of the Java strain are similar to those of *An. dispar*, *An. notanandai* and *An. maculatus* (*s.s.*) in having a very limited amount of pericentric heterochromatin, but these species have large submetacentric sex chromosomes. Therefore, the metaphase karyotype appears to be diagnostic for the Java strain.

The results of the cross-mating experiments clearly indicate genetic incompatibility between *An. maculatus* from Java and *An. maculatus* (*s.s.*) from Thailand. Hybrid males are completely sterile, while hybrid females appear to be normal. This may indicate a moderately high divergence between the two taxa. Greater divergence was observed when hybrid females and males exhibited both sterility and unviability, such as the crosses between *An. rampae* and *An. pseudowillmori*/*An. dravidicus* [8]. Unfortunately, it was not possible, due to unavailability of colonies, to determine relative levels of divergence from other species of the Maculatus Group. However, the results of the phylogenetic analyses of ITS2 and *cox*2 sequences conducted during the current study suggest that the Java strain, based on lowest K2P genetic distances, is more closely related to the Philippine *An. dispar* and the continental *An. maculatus* (*s.s.*) Additionally, based on morphological observations, the Java strain more closely resembles *An. dispar*, *An. greeni* and *An. maculatus* (*s.s.*) than the other members in the Maculatus Group, suggesting that the Java strain, *An. dispar* and *An. greeni* have a closer affinity with the Maculatus Subgroup than with the Sawadwongporni Subgroup as reported previously [10, 40].

## Conclusions

In conclusion, based on the morphological, cytogenetic, cross-mating and molecular evidence gleaned from the current study, *An. maculatus* in Java is not conspecific with continental *An. maculatus* and is a distinct species of the Maculatus Group, which is far more diversified than previously thought. This finding raises a question about whether there may be other undescribed species of the group on other islands of the Indonesian Archipelago, in particular Borneo, Sumatra and Sulawesi. Employing a combination of approaches to include traditional morphology, genetic and molecular methods of investigation is essential for recognizing and distinguishing closely related and morphologically similar species.
